# The Present Status of Surgery of the Brain and Spinal Cord*Read before the State Medical Association of Texas, at Amarillo, Texas, May 9-11, 1911. Reproduced from Virginia Semi-Monthly Medical Journal.

**Published:** 1911-11

**Authors:** A. W. Fly

**Affiliations:** Galveston, Texas


### Abstracts and Selections.

### The Present Status of Surgery of the Brain and Spinal Cord.*

A critical review of the progress of surgery of the brain and spinal cord impresses one with the fact that the work of the past decade has been vast and brilliant, yet as it is not my purpose to detract in the least degree from the high measure of praise and commendation due to surgery and surgeons of the decade that is gone, still we must admit the explorer of all new lands, unguided by experience, commits error apparent to his retrospection. So surgery, in its new work, has with enthusiasm advanced some doctrines which its present wisdom and experience must lead it to discard. The collection of data must precede the classification and establishment of any true science or enunciation of fixed principles arising therefrom. These years which have gone before, therefore, have been invaluable in the collection of vast elements of advanced truth.

*Read before the State Medical Association of Texas, at Amarillo, Texas, May 9-11, 1911. Reproduced from Virginia Semi-Monthly Medical Journal.

Recent years must be considered as time most wisely spent in the critical analysis of the facts already adduced, and in the deduction of sound principles for future work. This is the time of reckoning, and we should now be able to, in a measure, weigh our discoveries, and ascertain the true relation of surgery to the problems of human life and happiness. The result of these deliberations has demonstrated to us many excesses of our zeal, and led to a decided trend toward conservatism in surgery, not that conservatism, however, which but masks the face of ignorant inactivity, but a truer and higher conservatism which puts life first, then function and, finally, form.

Today, we are emerging from a tidal wave of brain surgery. There is scarcely a pathological condition to which the brain is subject that has not been submitted to the trial of operative surgery for its relief. Medical skill had for years exercised its best efforts for the cure, or control, of the diseases of cerebral origin, and, in a considerable number of cases, with only limited success. With great expectation and hope, therefore, the profession turned its thought to the possibility of relief by surgical measures. In this department of work have been enlisted the services and talents of many of the most eminent neurologists throughout the world. These laborers have brought to us knowledge of inestimable value.

Cerebral localization and an accurate understanding of much of the physiology and physiological anatomy of the various portions of the brain are achievements which have placed medicine upon a scientific and an accurate basis in the diagnosis of cerebral pathology, and shall forever stand as a brilliant record to the credit of brain surgery. Our efforts, viewed from a therapeutic standpoint, have not been so gratifying. With the possibility of relieving the distressing and heretofore largely hopeless cases of epilepsy, for instance, how eagerly and even enthusiastically it was embraced! The theory that all epilepsy of cerebral origin was attributable to local excitation, and the removal of the offending cause would insure a disappearance of its ensuing clinical manifestations, seemed indeed logical, both in its premises and its conclusions. All forms and degrees of epilepsy, whether general or focal, have been, therefore, subjected to varied operative procedures. The temporary benefit following nearly all operations, for a short time led us to believe that we finally had found the happy solution of this vexatious problem. But, for permanent results, we have learned that our power was limited in many cases.

In general epilepsy, it was soon demonstrated that little is to be expected from surgical intervention. This, however, has occasioned no great surprise, as little rational hope was ever entertained for this class of cases. Even in the focal, cortical epilepsy, however dependent upon distinct and localized lesion, susceptible of removal, the history of our work shows some lack of the permanency in results. As Eulenberg says: "By the excision of an area of the cerebral cortex, the attacks may be made to stop for some time, but they are apt to return after a while." Von Bergman asserts that "Only those cases of cortical epilepsy are cured by trephining which are due to tumors, especially cysts, which are often the result of intra-meningeal exudations, due to traumatism over or within the circumscribed motor area." Nancrede also affirms that his experience, like that of Horsley, Keen and other surgeons, has demonstrated a lack of permanency in results, and looks upon the removal of the discharging lesion in cortical of Jacksonian epilepsy as a merely palliative procedure. He further observes that "The earlier the operation is done after the disease has become established, the longer the immunity," and adds that: "It is possible that if the trephining were resorted to early, the operation would probably prove curative, if a reliable method were used to prevent the inevitable scar and the adhesions between the brain and its meninges." The universal experience, however, demonstrates that after degeneration of nerve substance has set in, it is too late for a permanent cure by surgery. We may remove the primary cause, but the degeneration, which is secondary, and the efficient cause of epilepsy, is just a little beyond the control of present methods. One vastly important observation, however, has been impressed upon the surgeon's mind by his experience in the surgery of epilepsy, viz: that a large percentage of the cases of epilepsy are due to remote traumatism of the skull, either unrecognized at the time, or, if recognized, improperly handled. The significance of this observation should not be overlooked in dealing with any injury of the head, and I confidently believe that the true conservative surgery of traumatic epilepsy is radical surgery of the trauma. With Lanphear, I can quite agree in the aphorism: "The way to cure traumatic epilepsy is to prevent it."

The operative treatment of imbecility and idiocy has even been less successful than in epilepsy. As Dana has aptly said: "This operation was originally devised on the theory that, by cutting open the skull of the microcephalic child, opportunity was given the brain to grow." This theory, which was never substantiated by facts, and never held by any experienced neurologist, has been, of course, overthrown. What the operation does is this: it has a profoundly disciplinary effect upon the idiot. The operation of craniotomy upon children in institutions attracts attention of nurses and all of the medical officers, and children get more care and more stimulating words of help in various directions. I repeat, therefore, that it is, in my opinion, largely due to its pedagogic influence, that so many cures are effected in the case of children. I believe, however, in view of the hopeless and practically helpless condition of these unfortunates, their lives being a burden to their friends and a mere blank to themselves, I believe, I say, that surgery has been a veritable Godsend to these children. Yet I fear that we are prone to lose sight of the moral side of this question. Senn calls attention to this in very forcible language: "I am free to confess that I have never been able to muster my courage to attack the skull of a poor microcephalic child, because I have always regarded the operation as useless in promoting brain development. The responsibility of the surgeon is not limited by the defective mental development of the child, nor the importunity of the parents demanding the operation at all hazards. The surgeon should stand guardian over such a charge, mindful of the limits of the art of surgery. Have we a right to estimate human happiness? The driveling idiot has many enjoyments that you and I know nothing of. His responsibilities to God and man are limited, and his existence on earth is a long, happy dream, which only ceases when the soul leaves the imperfect body and returns from whence it came—where mental distinction is unknown." The rule for operative interference in these cases is covered by Mr. Champneys. He says: "The number of cases in which operation is justifiable, or desirable, must be determined, generally speaking, by the ascertained risks of the disease against the risks of the operation; the former must exceed the latter before the operation should be justified." Life must be reckoned as the first object of our care, to be hazarded only on most mature indication.

In spinal cord surgery, some very brilliant advances have been made. The surgery of this part of the body has been wonderful, when we consider the short time in which it has all been accomplished. Just what is the relation of surgery to the spinal cord is, in my opinion, still an open question. The uncertainty of the differentiation of the various forms of its disease, its manifold aspects, clinically, render a scientific and fixed stand impossible in the light of our present knowledge. The mass of clinical observations in the treatment of the spinal cord which have been scientifically recorded have been from those treated surgically. A corresponding amount of evidence regarding the non-surgical treatment is not obtainable. True, the spinal cord was treated medically for many years, before surgery of this part of the body was ever introduced. At that time, however, the pathology of the conditions was practically unknown, and the differentiations were so obscure that accurate data was rarely had, and retrospective diagnosis and records were not entirely to be depended upon.

Thus, it will be seen that, in surgery, we are sifting the gems from the sands we have shoveled in the past years, and separating the true from the false doctrines in modern surgery. And it is becoming to the medical profession to view our present status and utilize our mass of accumulated knowledge, rather than wander into new fields of investigation. But few of us ever originate anything new. Science is studied abstractly, else perversions may arise. It is thus step by step "till the end is reached—the crowning point—a new idea. The utilitarian age comes in and applies."

The modern hospital is the outgrowth of this idea, made safe by science, from a surgical standpoint, far safer than any home. Hospitals tend to concentrate talent. Talent, when brought in contact, tries the old, and leads the new. Hence, we look to those for our gleanings. Still, I am very willing to admit that many new impulses are given by individual exertion. For example: Sims gave a new impulse to gynecology when he used an iron spoon from a kitchen; out of it grew Sims' speculum, out of which grew many valuable operations to return suffering women to the world.

Every well regulated town should have a hospital, however small, many a life might be saved. What would a man give in exchange for his life? The city surgeon will scarcely appreciate the difficulties surrounding his country cousin. The laboratory led the way to true progress. No organ in the body can escape the trained surgeon's knife.

The old hospitals were merely hotbeds for all forms of bacterial life; deadly germs lurked in every nook and corner. Since laboratories have shown this habitant, how different now! Medicine as a science is but an infant, and can trace its lineage only a few years. Superstition through long ages held progress in bondage. The most natural things in the world are simple laws that govern organic life—plant life and animal life—reaching up to the crowning work of God—man life. All obey the same laws of change. The surgeon has long since ceased to be called an operator, under the guide of a more learned man, but now must add unto his skill as an operator a knowledge of all life's laws, both health and disease. This is the modern surgeon.

In this connection, I wish to speak of the lack of general recognition that is given to the medical profession for the discoveries made by them in the field of brain surgery, as well as other lines. A distinguished statesman from New York once said: "We can not but think that the discoveries and improvements in medical practice, which are now being enjoyed, were dearly bought, if the medical profession, in their onward march, have left behind their sense of civic obligation, and their interest in the general public welfare. We can not accuse you of utter neglect of your duty to the country, yet we can not keep out of mind the suspicion, if your professional work in exposing evils were more thoroughly supplemented by labor in the field of citizenship, these evils would be speedily corrected. If members of our profession were oftener found in our national and State assemblies, ready to advocate the adoption of new discoveries which you have demonstrated to be necessary, and to defend your profession against impracticability, the prospects of bestowing upon your fellow men the ripened results of your professional labors would be brighter and nearer."

I do not believe it detracts from a man's professional usefulness for it to be known that he has a knowledge of something besides medicine. I do not believe it detracts from his ability to pursue scientific investigations for him to rest himself from his work by occasionally thinking of the affairs of State, literature and business. I do not believe that the medical man who confines himself to medicine, or to one branch of it, and who shuts himself in from the world and from the consideration, in moderation of his duties, is as successful a practitioner, as successful an investigator or as successful in his business as the man who is broader in his views. Exclusive working in a single line can not but have a narrowing effect. We need in surgery not only science, but philosophy and business. I am a firm advocate of dignity, and have always been in a position to appreciate the duties of a doctor, but my observation has been that we physicians pay too little attention to citizenship, and too little attention to the business part of our work. The consequence is, that the doctor, while he may be respected in his community for his professional attainments, has little stand in the business world, and his views have little weight with the public. I believe that the medical man who adds patriotism and business qualifications to his professional knowledge is a better doctor, a better citizen and of more value to his locality than the recluse who prides himself on knowing nothing of affairs, and would sooner live on crusts than have it known that he would venture to let it be known that he would demand his rights. Throughout the country, the large cities expend vast sums of money in caring for the unfortunates in hospitals and asylums, patients who require the best that advanced surgery and medicine can give. I contend that no men are as capable of proportioning that money in the channels in which it will do the most good, and be most economically expended, as educated men with business qualifications.

In our professional walk, we see the world as it is, not as it should be. We are in closer touch than any other calling with humanity, and get our experience of the condition of citizens from the poorest as well as from the richest. We thus have a special advantage in forming opinions on many economic questions of the day not enjoyed by others. Let us, then, add to this the business qualification of the average layman, let us use every effort to convince an enlightened community that we no longer are a part of a mystic cult, but that we are men like other men. We have a business founded upon science; let us perform our duties to our country as citizens, and exercise the business tact we owe our families and to ourselves, and then we will occupy the position before the public that belongs to the ideal physician.

So, in conclusion, I will say that, in the surgery of the brain and spinal cord, the surgeon has been truly marvelous. Yet, we have made mistakes; no true progress is ever made in life without mistakes along the way. But we, as physicians, must be aggressive if we would have the world reap the benefits of our progress.—A. W. Fly, M. D., Galveston, Texas.

